# Associations of the COVID-19 pandemic with older individuals’ healthcare utilization and self-reported health status: a longitudinal analysis from Singapore

**DOI:** 10.1186/s12913-021-07446-5

**Published:** 2022-01-14

**Authors:** SangNam Ahn, Seonghoon Kim, Kanghyock Koh

**Affiliations:** 1 School of Public Health, University of Memphis, Memphis, TN USA; 2 School of Economics, Singapore Management University, 90 Stamford Road, Singapore, 178903 Singapore; 3 Department of Economics, Korea University, Seoul, South Korea

**Keywords:** COVID–19, Pandemic, Healthcare utilization, Healthcare spending, Self-reported health status

## Abstract

**Background:**

The COVID–19 pandemic has challenged the capacity of healthcare systems around the world and can potentially compromise healthcare utilization and health outcomes among non-COVID–19 patients.

**Objectives:**

To examine the associations of the COVID-19 pandemic with healthcare utilization, out-of-pocket medical costs, and perceived health among middle-aged and older individuals in Singapore.

**Method:**

Utilizing data collected from a monthly panel survey, a difference-in-differences approach was used to characterize monthly changes of healthcare use and spending and estimate the probability of being diagnosed with a chronic condition and self-reported health status before and during the COVID-19 outbreak in 2020.

**Subjects:**

Data were analyzed from 7569 nationally representative individuals from 2019 January and 2020 December.

**Measures:**

Healthcare utilization and healthcare spending by medical service categories as well as self-reported health status.

**Results:**

Between January and April 2020 (the first peak period of COVID-19 in Singapore), doctor visits decreased by 30%, and out-of-pocket medical spending decreased by 23%, mostly driven by reductions in inpatient and outpatient care. As a result, the probability of any diagnosis of chronic conditions decreased by 19% in April 2020. The decreased healthcare utilization and spending recovered after lifting the national lockdown in June, 2020 and remained similar to the pre-pandemic level through the rest of 2020.

**Conclusions:**

Middle-aged and older Singaporeans’ healthcare utilization and the diagnosis of chronic conditions substantially decreased during the first peak period of the COVID-19 outbreak. Further studies to track the longer-term health effect of the pandemic among non-COVID-19 patients are warranted.

## Introduction

The novel coronavirus disease (COVID–19) has disrupted healthcare systems around the world. It is a direct health threat not only to patients who are infected but also to those who are not, due to strained healthcare capacity and fear of the infection. Many non-COVID-19 patients delayed or cancelled necessary healthcare [1]. As a result, healthcare utilization significantly dropped globally, e.g., by 38% in severe heart attack patients treated in nine major hospitals in the U.S. [2], and 64% in pediatric emergency department (ED) visits in Germany [3].

Reduced or delayed healthcare utilization during the pandemic can have detrimental health consequences. For instance, patients may suffer from delayed routine care, diagnoses, and elective procedures, while halting clinical trials could have long-term negative effects on medical research [4, 5]. In particular, restrained healthcare services can affect older adults with chronic conditions more severely [6, 7]. When older adults express fear and anxiety because of the pandemic, they are encouraged to self-isolate for an extended period of time [8]. This could significantly generate serious health consequences via decreases in healthcare utilization and spending. Few studies have demonstrated how healthcare utilization, out-of-pocket medical spending, and health outcomes change during the COVID–19 pandemic, primarily because of the lack of high-quality individual-level longitudinal data [9]. We fill this gap in the literature by using nationally representative individual-level monthly panel data from the Singapore Life Panel (SLP), collecting rich information, such as healthcare utilization, healthcare spending, and health outcomes, from Singaporeans aged 56–76 and their spouses. We specifically examine the extent to which these outcomes of interest evolved before and during the pandemic among older individuals where an efficient national single-payer healthcare system along with a mandatory health savings account is operated [10].

Singapore reported the first COVID–19 case in the early period of the pandemic on January 23, 2020. By mid-April, the number of confirmed cases exploded due to contagion in high-density dormitories of low-wage migrant workers. In the early stage of the pandemic, the government started to provide subsidized treatments and medications to patients with respiratory symptoms via “public health preparedness clinics.” Concerned with the spike in confirmed cases, the Singapore government imposed a set of nationwide partial lockdown policies, called the circuit breaker (CB), from April 7 to June 1. People could still take public transport and leave their homes, but social gatherings were prohibited. While “essential” services (e.g., urgent healthcare, transportation, groceries) were still allowed to operate, workers in “non-essential” services were required to work from home and all schools remained closed. In addition, the government temporarily allowed patients to use the medical savings account balance (called Medisave) to pay for telemedicine services (e.g., online consultations and chronic disease management). Previously, the medical savings account balance was designed to pay for relatively severe conditions. When the lockdown measures were lifted in early June, the number of COVID-19 cases decreased substantially and has become fewer than 10 cases a day by the last quarter of 2020. For example, in December 2020, there were only 13 monthly cases confirmed, and the same trend continued in early 2021.

In this study, we investigate changes in healthcare utilization, out-of-pocket medical costs, and perceived health outcome among older Singaporeans before and during the COVID-19 outbreak, using the nationally representative, high-frequency panel data.

## Study data and methods

Since July 2015, the SLP has surveyed nationally representative cohorts of Singapore residents currently aged 56–76 and their spouses on a monthly basis. Survey participants were randomly sampled by the Singapore Department of Statistics based on probability proportional to the population size [11]. Around 8000 individuals participated in the survey with little attrition over the last 6 years. Between January 2019 and December 2020, the attrition rate was 6%. We believe that the SLP is an ideal dataset when studying the association of COVID–19 with healthcare utilization and self-reported health. The SLP tracks individuals’ healthcare utilization, healthcare spending, and self-reported health status as well as other individual and household characteristics on a monthly basis before and during the pandemic. Additionally, survey participation has not been interrupted by COVID–19 because the SLP is an internet-based survey. For respondents who do not have access to the Internet or have difficulty in doing the online survey, the SLP conducted telephone interviews. We used the SLP data covering up to December 2020.

**Fig. 1:**
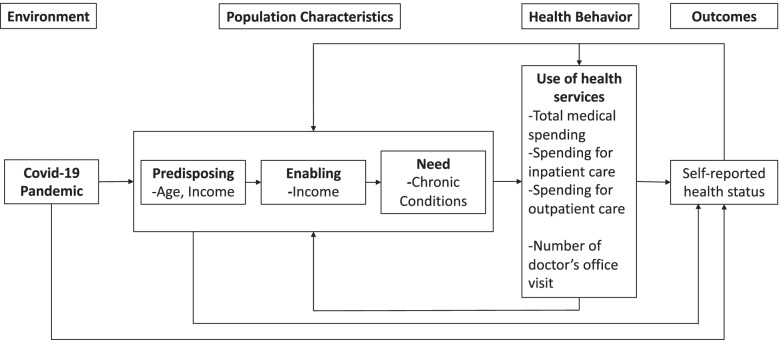
Conceptual Framework Based on Anderson Behavioral Model of Health Services Use

The Andersen model of healthcare use has guided our approach to selecting variables and comprehending our findings [12]. As the model posits (Fig. 1), forgone healthcare (health behavior) can be the function of COVID–19 (environment) and population characteristics (e.g., age [predisposing], income [enabling], chronic conditions [need]), which all may affect health outcomes (perceived health status).

We measured healthcare utilization and healthcare spending as follows. First, we constructed a binary indicator of whether an individual met a medical doctor each month. Second, we created a dummy variable by assigning “1” if a respondent has been told by a doctor that he or she has any health conditions, such as hypertension, diabetes, cancer, heart problems, stroke, arthritis, or psychiatric problems, and “0” if the respondent has not been diagnosed with any. Third, we constructed total healthcare spending by summing up six medical service categories: 1) inpatient care including hospital and nursing home care; 2) outpatient care including visits to doctors, traditional Chinese physicians, physiotherapists, and psychologists, eye care and dental service fees, and laboratory tests; 3) prescription drugs; 4) other drugs and medical products (e.g., Chinese medicine, wheelchairs); 5) health insurance premiums; and 6) hiring home nursing services. All monetary units are expressed in 2019 Singapore dollars (S$).

We measured perceived health status through a single question on self-reported health status. Respondents were asked to rate their health status on a 5-point scale of “excellent,” “very good,” “good,” “fair,” or “poor.” [13] We constructed a dummy variable by assigning “1” if a respondent’s self-reported health status is “good,” “very good,” or “excellent,” and “0” if otherwise.

### Analytic approach

We used a difference-in-differences (DID) method by comparing monthly changes to estimate the associations of the COVID–19 outbreak with individuals’ healthcare use and spending, and perceived health status between 2020 and 2019 (“two seasons” hereafter). Specifically, we estimated Eq. (1):

1 $$y_{i,t}=\beta_0+\beta_1Season_t+{\textstyle\sum_{k\neq Jan}}\beta_k1\left[\mathit{Mt}h_t=k\right]Season_t+\mathit{Mt}h_t+\lambda_i+X'_{i,t}\gamma+\epsilon_{i,t}$$

where *y*_*i*, *t*_ represents healthcare utilization, healthcare spending, and health status of individual *i* in month *t*. *Mth*_*t*_ are month dummies. January serves as the reference month because Singapore’s first COVID-19 case was confirmed on January 23, 2020. *Season*_*t*_ is a dummy variable whose value is “1” if the observed period is between January 2020 to December 2020, and “0” otherwise. *λ*_*i*_ denotes individual fixed effects. *X*_*i*, *t*_ includes age, age squared, marital status, and household size. *β*_*k*_ s are the parameters of interest, which may capture associations between COVID–19 and dependent variables in each month compared to those in January. For statistical inference, we calculated standard errors clustered at the individual level to adjust for serial correlations of dependent variables within individuals.

To show the association between the COVID-19 pandemic and our outcomes of interest, we compare monthly trends of these outcomes between 2019 and 2020. Statistical analysis was conducted using STATA/MP 16 (StataCorp, College Station, Texas, USA).

## Results

Table 1 describes the socio-demographic breakdown of the study population as of January 2020, the month before the COVID-19 outbreak in Singapore (*N* = 7569). The average age of study participants was 63.2 (standard deviation [SD] = 6.4 years). There were more female (53%) than male (47%) participants. 23% graduated from the primary school level, 41% from secondary, and 36% from tertiary. Out of all respondents, 87% are ethnic Chinese and about 79% are married. On average, study participants had 2.91 children (SD = 1.13) and 2.6 members (SD = 1.39) in their households.

**Table 1:** Sociodemographic distribution of respondents to the survey before the COVID-19 outbreak in Singapore, January 2020 (*N* = 7569)

Variables	Mean (±SD) or N (%)
Age	63.2 (±6.40)
Sex	
Male	3561 (47%)
Female	4008 (53%)
Education	
Primary	1721 (23%)
Secondary	3143 (41%)
Tertiary	2705 (36%)
Ethnicity	
Chinese	6548 (87%)
Non-Chinese	1021 (13%)
Marital status	
Married	5962 (79%)
Unmarried	1607 (21%)
Number of children	2.91 (±1.13)
Number of household members	2.60 (±1.39)

Source. Authors’ analysis of data from the Singapore Life Panel Survey January 2020 Wave

Note. *SD* Standard Deviation

**Fig. 2:**
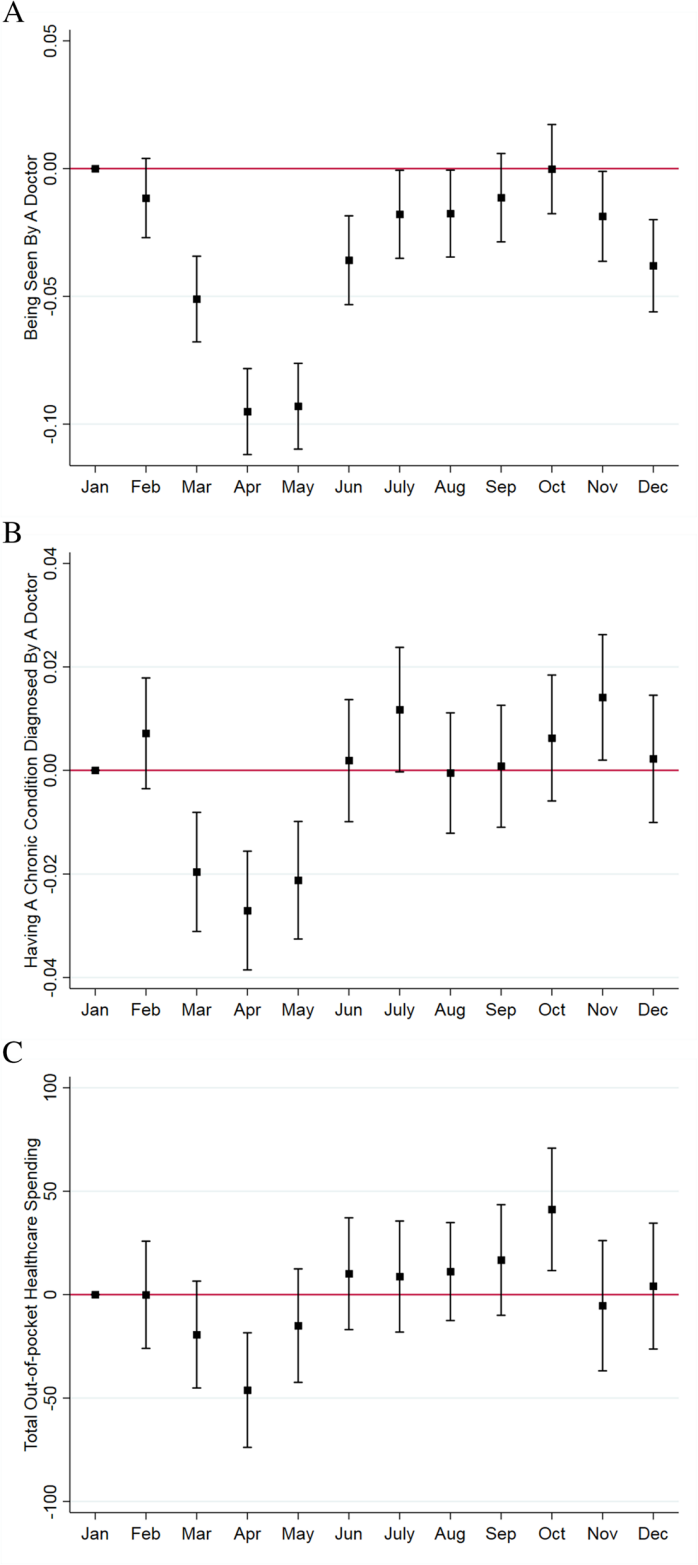
Monthly changes in healthcare utilization, diagnosis of chronic conditions, and out-of-pocket medical spending in Singapore, 2019–2020. **A**. The share of respondents seen by a doctor. **B**. The share of respondents having a chronic condition diagnosed by a doctor. **C**. Total out-of-pocket healthcare spending (in S$). Source. Authors’ analysis of data from the Singapore Life Panel Survey. Notes. Square dots represent point estimates and caps indicate 95% confidence intervals using Eq. (1). Standard errors are clustered at the individual level and corrected for heteroskedasticity

Figure 2 (Panel A) demonstrates that individuals were less likely to visit medical doctors during the COVID-19 outbreak. The DID estimates show that the share of survey participants who visited a medical doctor sharply decreased to around 10 percentage points when the lockdown was implemented for 2 months in April and May 2020. The estimates are statistically significant at the 5% level. After lifting the lockdown in early June 2020, the likelihood of visiting any doctor increased approximately by 6 percentage points. From July through December 2020, the share of any doctor visit increased to almost 30%, but it was still lower than its pre-pandemic level.

Figure 2 (Panel B) demonstrates that study participants were less likely to be diagnosed with a chronic condition during the peak period of the COVID–19 outbreak (March–May 2020). The share of respondents diagnosed with chronic conditions decreased by 2.7 and 2.1 percentage points in April and May 2020 respectively, which corresponds to a 19% reduction from the January 2020 average. This dip quickly recovered from June 2020 onward when the nationwide lockdown was lifted. As a result, the share of respondents diagnosed with chronic conditions recovered to its pre-pandemic level.

Figure 2 (Panel C) demonstrates that study participants were less likely to spend on medical care during COVID–19. The medical expenditures decreased by S$46 in April (a 23% reduction from the January 2020 average), and it rebounded to the pre-pandemic level in June 2020 onward.

**Fig. 3:**
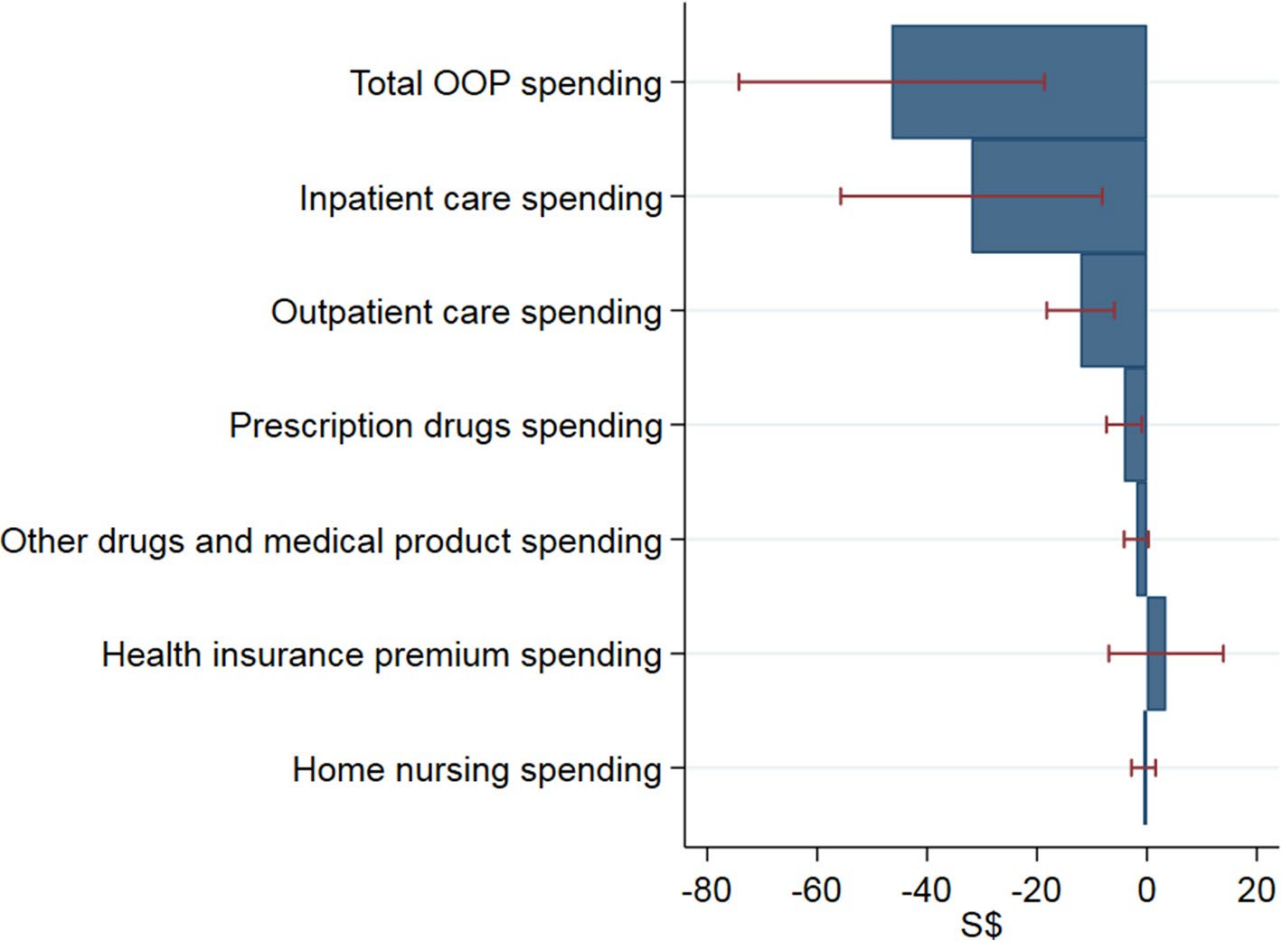
Changes in medical care spending by type of medical service between January 2020 and April 2020. Source. Authors’ analysis of data from the Singapore Life Panel Survey. Notes. Caps indicate 95% confidence intervals. Standard errors are clustered at the individual level and corrected for heteroskedasticity. OOP = out-of-pocket

We further examined changes in out-of-pocket healthcare spending by type of medical services such as inpatient care, outpatient care, prescription drugs, other drugs and medical products, health insurance premium, and home nursing. Figure 3 shows the estimated decrease in out-of-pocket healthcare spending by type of medical services between January 2020 and April 2020. It indicates that reductions in inpatient care and outpatient care account for 69 and 26%, respectively, of the total out-of-pocket healthcare spending reduction during the peak month of the pandemic in Singapore. These estimates are statistically significant at the 5% level.

**Fig. 4:**
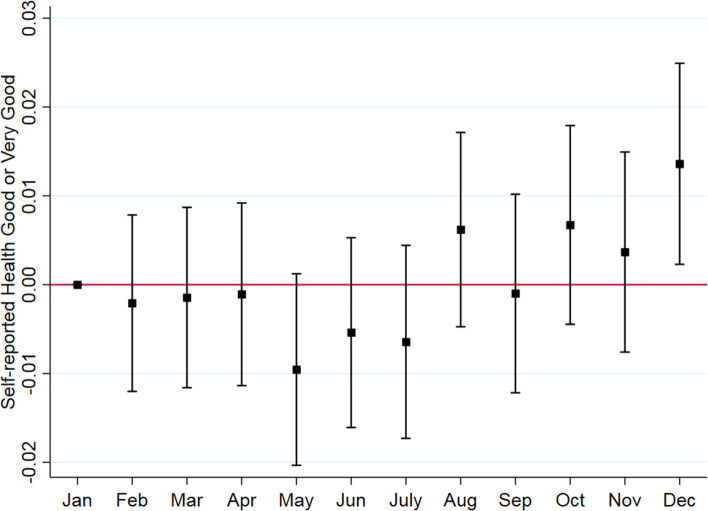
Monthly changes in self-reported health status in Singapore, 2019–2020. Source. Authors’ analysis of data from the Singapore Life Panel Survey. Notes. Square dots represent point estimates and caps indicate 95% confidence intervals using Eq. (1). Standard errors are clustered at the individual level and corrected for heteroskedasticity

Figure 4 displays DID estimates of the effects of COVID-19 on self-reported health status. It indicates little evidence that COVID-19 increased the proportion of respondents who reported having good, very good, or excellent health status between the 2 years. The estimates are close to zero and statistically insignificant.

## Discussion

Previous studies have mainly documented snapshots of healthcare utilization [14, 15] and perceived health status [16] in the midst of the pandemic. To extend the literature, we provide comprehensive evidence on the changes in healthcare utilization, healthcare spending, and perceived health status among older Singapore residents during the COVID-19 pandemic using the individual-level monthly panel data.

After the outbreak of COVID–19, older Singaporeans reduced their healthcare utilization by 23% in terms of total out-of-pocket healthcare spending. During the pandemic, there was a similar decrease in healthcare utilization in the U.S.: 32–40% in ED radiology volumes [17] and 49% in acute ischemic stroke patients [18]. Delaying healthcare is associated with longer hospital stays and poor health outcomes in the future [19]. However, based on the SLP’s self-reported health status data up to December 2020, we find no evidence of worsened health outcomes although healthcare utilization was significantly reduced during the peak period of COVID-19 in April and May 2020.

The reduced healthcare utilization can be related to both less out-of-pocket spending on healthcare services and fewer diagnoses of chronic conditions. Regarding out-of-pocket medical spending, few studies tracked changes in the spending during an epidemic [9, 20], especially by medical service type. This study found that reduced healthcare spending during the pandemic was primarily driven by the decreased inpatient (69%) and outpatient care spending (26%), which is similar to the significant reductions in inpatient care (35%) and outpatient care (24%) observed during the peak of SARS [21]. Patients’ spending reduction in inpatient care was expected when many elective surgical procedures were postponed subsequent to the WHO’s declaration of COVID-19 as a pandemic [22, 23]. Reduced spending in outpatient care was expected to occur when patients and physicians cancelled their non-essential visits due to the pandemic. In addition, reduced healthcare spending can be linked to the decreased number of diagnoses of chronic conditions (e.g., cancer, diabetes, stroke).

From a healthcare provider perspective, reduced out-of-pocket spending and disrupted healthcare systems can have considerable financial consequences. When the Singapore government implemented a nationwide partial lockdown policy from April 7 to June 1, 2020, several complaints were reported from healthcare providers (especially dentists) fearing the loss of their patients and revenue [24]. In the U.S., hospitals and health systems reported financial losses amounting to $202.6 billion from March 1 to June 30 in 2020 [25], and it was estimated that primary care practices would lose US$67,774 in gross revenue per full-time physician in 2020 [26]. Furthermore, the financial consequences are dire when it is difficult to forecast when patients can return to physicians’ offices and hospitals (e.g., for non-emergent surgery or regular checkup) without fear of contracting the virus [27]. In addition, healthcare delivery systems will be further challenged when patients who delayed care during the pandemic ultimately make ED visits [28].

Unlike the Andersen Model we employed in this study, self-reported health status did not change significantly during COVID–19 due to following three reasons. First, Singaporeans have maintained relatively good health before and during the pandemic, and have an efficient national single-payer healthcare system. Singapore has a long life expectancy at birth (83.8 years in 2020) as well as a low fatality rate from COVID-19 (0.05%), compared to the world average (2.3%) as of November 30, 2020 [29]. Second, healthcare utilization decreased only during the peak of COVID-19, and quickly recovered to its pre-COVID-19 level. Finally, patients may have only reduced nonessential healthcare, which could have negligible health impacts during the pandemic. The self-reported health status appeared to improve in December 2020. One possible explanation is that there was an improvement in subjective well-being with respect to economic situations during the same period as the economy began to rebound [30]. Since subjective well-being is positively associated with individuals’ health status, we argue that improved subjective well-being may translate into better health status.

We acknowledge limitations of this study. First, we used self-reported survey data. Thus, the collected information on healthcare utilization, healthcare spending, and perceived health status could be subject to measurement errors. Second, our study is based on observational data and thus there could be other plausible explanations for changes in healthcare utilization, healthcare spending, and perceived health status during the pandemic. Third, we cannot separate essential and nonessential care due to the data limitation. It would be meaningful to investigate whether patients reduced mainly nonessential care without having negative health impacts during the pandemic. Finally, we focused on short-term impacts of COVID-19; thus, further studies are warranted to track the long-term health effect of the pandemic among non-COVID-19 patients.

## Conclusion

The current study provides a unique perspective related to changes in healthcare utilization, healthcare spending, and perceived health status among older adults during the COVID-19 pandemic. Our findings have the following implications. First, government-imposed restrictions should be carefully implemented to avoid interrupting “essential” healthcare services among non-COVID–19 patients. Second, as the pandemic is prolonged, governments should continue monitoring the long-term health effects of non-COVID–19 patients, especially those who have existing health conditions and delayed healthcare visits. Although we find no evidence of short-term health impact, it is possible that adverse health consequences of delayed healthcare visits emerge later. Lastly, patients should be “activated” to self-care for their chronic conditions, especially older adults with multiple chronic conditions, as their access to healthcare can continue to be limited until the pandemic is over.

## Data Availability

The data that support the findings of this study are available from the Centre for Research on Successful Ageing (ROSA) at Singapore Management University but restrictions apply to the availability of these data, which were used under license for the current study, and so are not publicly available. Data are however available from the authors upon reasonable request and with permission of the ROSA.
